# Ocular and systemic risk factors associated with recurrent disc hemorrhage in primary open-angle glaucoma

**DOI:** 10.1371/journal.pone.0222166

**Published:** 2019-09-16

**Authors:** Bo Ram Seol, Jin Wook Jeoung, Ki Ho Park

**Affiliations:** 1 Department of Ophthalmology, VHS Medical Center, Seoul, Korea; 2 Department of Ophthalmology, Seoul National University Hospital, Seoul National University College of Medicine, Seoul, Korea; University of Warmia, POLAND

## Abstract

**Purpose:**

To evaluate the risk factors associated with recurrent disc hemorrhage (DH), defined for the present study as at least 3 occurrences of DH in primary open-angle glaucoma (POAG).

**Methods:**

A total of 178 POAG patients (89 eyes showing at least 3 occurrences of DH and 89 age-matched control eyes with a minimum of 10 years’ follow-up without DH) were included in a retrospective, case-control study. Ocular factors were evaluated by a retrospective chart review, and systemic factors were evaluated by a telephone survey. Associations between factors and recurrent DH were investigated by logistic regression analysis. The Kaplan–Meier survival analysis and Cox proportional-hazards regression models were used to evaluate glaucoma progression and to identify the factors predictive of glaucoma progression.

**Results:**

Univariate regression analysis revealed the association of recurrent DH with low baseline intraocular pressure (IOP) [odds ratio (OR), 0.88; 95% confidential interval (CI), 0.80–0.98; P = 0.014], lower percentage reduction of IOP (OR, 0.96; 95% CI, 0.93–0.99; P = 0.020), cold extremities (OR, 2.80; 95% CI, 1.03–7.60; P = 0.043), prone or lateral decubitus sleeping position (OR, 2.14; 95% CI, 1.13–4.03; P = 0.019), and sleeping disorders (OR, 2.33; 95% CI, 1.05–5.15; P = 0.037). Multivariate regression analysis revealed that a lower percentage reduction in IOP (OR, 0.96; 95% CI, 0.93–1.00; P = 0.046) increased the risk of recurrent DH. The control group exhibited a greater cumulative probability of non-progression than the recurrent DH group (P = 0.01, by log-rank test). The Cox proportional-hazards regression model showed that recurrent DH was associated with glaucoma progression [hazard ratio (HR), 1.88; 95% CI; 1.66–3.05; P = 0.01.

**Conclusions:**

Among the ocular and systemic factors, only lower-percentage reduction of IOP in POAG was associated with recurrent DH. DH recurrence is associated with glaucoma progression and may be dependent on IOP.

## Introduction

Previous studies have reported disc hemorrhage (DH) as an important risk factor for the development and progression of glaucoma [1, 2]. The Ocular Hypertension Treatment Study demonstrated that DH is a specific risk factor for the development of primary open-angle glaucoma (POAG), while the Early Manifest Glaucoma Trial, showed a significant association between DH and visual field (VF) progression [3, 4]. However, DH can occur not only in eyes with glaucoma but also in normal eyes. For example, the Blue Mountain Eye Study noted the presence of DH not only in 13.8% of participants with open-angle glaucoma (8% of patients with high-pressure glaucoma and 25% with low-pressure glaucoma) and 1.5% of patients with ocular hypertension but also in 1% of normal eyes [5].

Based on the possible occurrence of DH in normal eyes, we surmised that an evaluation of recurrent DH might be more appropriate, and certainly important, for glaucoma patients. Although the recurrence of DH is common, there are few studies that have investigated it. Those that have focused on the relationship between recurrent DH and glaucoma progression and reported controversial results [6–8]. Siegner et al.[6] and de Beaufort et al.[7] noted that there were no differences in the rates of glaucoma progression between recurrent-DH patients and single-DH patients. However, Ishida et al. [8] reported that recurrent DH eyes exhibited a more pronounced glaucoma progression than did single-DH eyes. In consideration of this correlation between recurrent DH and glaucoma progression, we considered an investigation into the risk factors associated with DH recurrence was necessary, as such knowledge could contribute to our understanding of the causative mechanisms behind it. To the best of our knowledge, no studies on the risk factors of recurrent DH has yet to be conducted. Therefore, we evaluated the risk factors associated with DH recurrence in POAG patients.

## Materials and methods

### Subjects

We conducted a retrospective chart review of POAG patients who had visited the glaucoma clinic at Seoul National University Hospital from January 2002 through March 2016. The study protocol, approved by the Institutional Review Board (IRB) of Seoul National University Hospital, adhered to the tenets of the Declaration of Helsinki. Due to the retrospective data analysis, the ethics committee waived off the requirement of informed consent. In addition, the IRB approved the use of oral consent for evaluating systemic factors by telephone survey. One glaucoma specialist (B.R.S.) explained the purpose of the survey to the participants, all of whom provided their oral consent.

All of the patients underwent a complete ophthalmologic examination including visual acuity (VA), intraocular pressure (IOP) measurement (Goldmann applanation tonometry), corneal pachymetry (Pocket II Pachymeter Echo Graph; Quantel Medical, Clermont-Ferrand, France), measurement of axial length (AXL; Axis II PR, Quantel Medical, Inc., Bozeman, MT, USA), slit-lamp examination, gonioscopy, Humphrey Visual Field Analysis (Carl Zeiss Meditec, Inc., Dublin, CA, USA) using the Swedish interactive threshold algorithm with a 30–2 standard program, and spectral-domain optical coherence tomography (SD-OCT) (Carl Zeiss Meditec, Inc.). Color disc stereo photography and red-free fundus photography were evaluated at intervals of 3–6 months, and VF tests at intervals of 6–12 months, according to patient status.

Participants who met the following criteria were included: (1) an open-angle glaucoma (OAG) diagnosis in 1 or both eyes at the first clinic visit, (2) no history of IOP-lowering treatment at baseline, and (3) treated with topical medications during the follow-up period. In addition, patients had to be older than 18 years of age and to have had a best-corrected visual acuity (BCVA) of 20/40 or better, good quality red-free photographs, and a reliable VF. Patients were excluded for any of the following reasons: retinal diseases that might affect the VF or the interpretation of red-free fundus photographs, including diabetic retinopathy and any history of other optic neuropathies. We further excluded those who had undergone glaucoma laser treatment (argon laser trabeculoplasty or selective laser trabeculoplasty) or glaucoma surgery (trabeculectomy or Ahmed valve operation). If both eyes were eligible for inclusion, one eye was selected randomly. All of the patients were treated for glaucoma at the discretion of the attending ophthalmologist (K.H.P.), who aimed to reduce baseline IOP by at least 20%.

A diagnosis of POAG was made in cases where a patient showed glaucomatous optic disc damage and corresponding glaucomatous VF defects, along with an open angle. Glaucomatous optic disc changes were defined on stereoscopic color disc photography as cup/disc (C/D) asymmetry between the glaucomatous and normal eye that was greater than 0.2, large cupping (>0.7 vertical C/D ratio), and neuroretinal rim thinning, notching, or excavation. Only reliable VF tests (false-positive errors < 15%, false-negative errors < 15%, fixation loss <20%) were included in the analysis. Optical Coherence Tomography (OCT) is widely used in clinical practice; however, various parameters can be used to diagnose glaucoma with OCT. For example, quadrant retinal-nerve fiber layer (RNFL) thickness, clock-hour RNFL thickness, ganglion cell-inner plexiform layer (GCIPL) thickness, the RNFL deviation map, and the GCIPL deviation map can all be used. As the purpose of this study was not to diagnose glaucoma, we considered it sufficient to diagnose glaucoma using disc photography, RNFL photography, and the VF test.

A narrow definition of recurrent DH was used, i.e., an eye showing at least 3 occurrences of DH during the follow-up period, with an interval between DH onsets of at least 6 months. Patients with eyes not showing any DH over the course of at least 10 years of follow-up were enrolled as part of the control group. The presence of DH was evaluated independently by 2 glaucoma specialists (B.R.S. and K.H.P.) and discrepancies between the two observers were resolved by consensus. Recurrent DH diagnosis was based on color disc stereo and red-free fundus photography and was defined as an isolated hemorrhage observed either in the optic disc or in the peripapillary retina and extending to the disc rim. Once the recurrent DH group was defined using the above definition, the control group was selected by age-matching.

If DH was detected, we re-examined the patient several months later to confirm that it had disappeared. We did not change the treatment based on a one-time occurrence of DH. In cases where accompanying glaucoma progression was observed, or recurrent DH occurred, we escalated the treatment.

### Ocular factors

We evaluated ocular factors by retrospective chart review. These factors included central corneal thickness (CCT), AXL, baseline IOP, IOP with medication (mean, fluctuation and maximum IOP, percent reduction of IOP), number of medications, VF global indices, and average RNFL thickness as measured by SD-OCT. Baseline IOP was measured prior to treatment and on every visit thereafter. Fluctuations of IOP was defined as the standard deviation during follow-up. The percent reduction of IOP was defined as follows: 100 * (baseline IOP–mean IOP during treatment) ⁄ baseline IOP.

### Systemic factors

Systemic factors were evaluated by telephone survey. This was done at the end of the follow-up period, and a standard phone script was used. The following systemic factors were investigated: diabetes, systemic hypertension, cardiac disease (ischemic heart disease or arrhythmia), the use of aspirin, anticoagulant and gingko extract, history of ocular operations and trauma, cold extremities, migraine, glaucoma family history, sleeping position (prone/lateral decubitus positions), yoga, sleep disorders (sleep apnea/snoring), constipation, carrying heavy loads, and wind-instrument playing. In the case of sleep apnea, self-reported snoring alone was not enough to confirm its presence, hence patients were asked if they had received a diagnosis of snoring. Constipation was defined as habitual constipation. Patients were checked for carrying heavy physical loads only if it was frequent and habitual. Wind-instrument playing was defined as that which required significant Valsalva maneuvers.

We did not perform detailed patient assessments on the status and severity of these systemic diseases, and patients who did not receive treatment after a diagnosis were excluded. Patients were also excluded if they had been previously treated for one of these diseases and normalized without any need for further treatment. In addition, any participants who were unsure of, or could not remember their status regarding any of these systemic factors, or experienced a significant change in their systemic disease status were excluded from the study. There is always the possibility that a patient neglects to mention or cannot remember what is listed on their medical record. Therefore, we also conducted a medical chart review on of all of the items included in the telephone survey. Ocular factors were judged by prioritizing hospital records. However, in the case of systematic factors, if the patient received treatment at our hospital and the patient's memory was different from the medical record, the hospital records were considered to be correct. Conversely, a patient’s memory was considered correct if it differed from their medical records when they had been treated at other hospitals.

### Glaucoma progression

Structural progression was determined by evaluating color disc and red-free RNFL photographs. Progressive optic disc changes (e.g., focal or diffuse rim narrowing, neuroretinal rim notching, increased cup-to-disc ratios, and adjacent vasculature position shifts) were determined by comparing serial disc photographs and regarded as indicating glaucoma progression. Changes in an RNFL defect were determined from serial RNFL photographs and defined as the appearance of a new defect or an increase in the width or depth of an existing defect. These were regarded as indicative of structural progression. Two observers (B.R.S. and J.W.J.), who were blinded to all other patient information, independently evaluated all photographs. In cases of disagreement, a third glaucoma specialist (K.H.P.) served as an adjudicator. Functional progression was determined by event analysis using the commercial guided progression analysis (GPA) software provided by the VF device. Functional progression was confirmed when at least three test points were flagged as having significantly deteriorated at the same test point locations in three consecutive fields (the software classifies VF progression as ‘likely progression’) [9, 10]. These changes also had to have been observed at the final visit.

### Statistical analysis

Comparisons of the baseline characteristics between the two groups were performed using the independent t-test for continuous variables and the chi-squared test for categorical variables. Univariate and multivariate logistic regression analyses with backward stepwise selection were used to identify the risk factors for recurrent DH. Variables with P < 0.10 in the univariate analysis, were used for multivariate analysis. The intergroup cumulative risk ratios of glaucoma progression were compared using the Kaplan-Meier survival analysis and log rank tests. The detection of initial progression was regarded as the end point in the survival analysis. The end of follow-up was determined as the point at which patients without progression were censored. The univariate and multivariate Cox proportional-hazards regression models with backward stepwise selection were used to identify risk factors for glaucoma progression. Variables with P < 0.10 in the univariate analysis were used for multivariate analysis. The proportional-hazards assumption was tested with the Schoenfeld residuals method and the assumption was not violated. Multicollinearity between factors was assessed by calculating the relevant variance inflation factors (VIF), to prevent multicollinearity. All the statistical analyses were performed using SPSS 18.0 (SPSS, Inc. Chicago, IL, USG) and R 3.5.1 (R Foundation, Vienna, Austria); P values less than 0.05 were considered statistically significant.

## Results

The study included 652 eyes from 652 POAG patients analyzed with color disc photography, red-free RNFL photography, and VF testing. Of these, 60 eyes were excluded because of poor quality red-free RNFL photography or VF testing. In addition, 58 patients who had retinal diseases or optic neuropathy and 18 patients who had undergone glaucoma laser treatment or surgery were excluded. Of the remaining 516 POAG patients, 166 showed 1 or 2 occurrences of DH, and 19 patients who had more than 3 occurrences of DH, but the intervals between them was less than 6 months, were also excluded. This left 331 POAG patients. Of these, 89 eyes from 89 POAG patients were included in the recurrent DH group and 89 eyes from 89 POAG patients were included in the age-matched control group. The other 153 patients did not exhibit any DH over the course of follow-up. However, they were not followed-up for more than 10 years because of intermittent visits, or follow-up loss and were excluded from the control group. A total of 178 eyes (89 recurrent DH eyes and 89 control eyes) from 178 POAG patients were included in this study. The patients’ demographic information and baseline characteristics are summarized in Table 1. The mean age was 64.18 ± 12.22 years, and the mean follow-up period was 126.94 ± 22.16 months. Patient age indicates the age at first visit. The recurrent DH group had a shorter follow-up period, more frequent follow-up visits and lower baseline IOP than the no-DH group (P <0.001, 0.001, and 0.010, respectively).

**Table 1:** Demographics and baseline data on 178 primary open-angle glaucoma patients.

	Total (n = 178)	Recurrent DH group (n = 89)	Control group (n = 89)	P-value
Age, mean (SD), years	64.18 (12.22)	64.55 (11.49)	63.81 (12.63)	0.683^a^
Male, number (%)	98 (55.06)	47 (52.81)	51 (57.30)	0.547^b^
Follow-up period, mean (SD), months	126.94 (22.16)	119.5 (25.83)	134.43 (14.38)	**<0.001** ^a^
Follow-up number, mean (SD), number	24.7 (4.79)	24.05 (5.28)	23.48 (4.32)	0.486 ^a^
Follow-up interval, mean (SD), months	5.60 (1.01)	5.30 (0.89)	5.86 (1.04)	**0.001** ^a^
CCT, mean (SD), mm	525.04 (39.55)	523.95 (32.52)	526.11 (40.01)	0.738 ^a^
AXL, mean (SD), μm	24.24 (1.27)	24.07 (1.07)	24.36 (1.24)	0.242 ^a^
Medication number (SD), number	1.40 (0.64)	1.47 (0.66)	1.29 (0.61)	0.060 ^b^
Baseline IOP, mean (SD), mmHg	15.59 (3.24)	14.92 (3.63)	16.40 (3.35)	**0.010** ^a^
Initial VF MD, mean (SD), dB	-4.24 (4.59)	-4.15 (5.34)	-4.33 (4.07)	0.807 ^a^
Initial VF PSD, mean (S), dB	5.54 (4.17)	5.35 (4.33)	5.72 (3.87)	0.543 ^a^
Average RNFL thickness, mean (SD), μm	88.60 (13.82)	90.31 (13.74)	87.27 (13.47)	0.225^a^

SD, standard deviation; CCT, central corneal thickness; AXL, axial length; IOP, intraocular pressure; VF, visual field; MD, mean deviation; PSD, pattern standard deviation; RNFL, retinal nerve fiber layer.

^a^ Independent t-test

^b^ Chi-square test; bolded values represent significance, P < 0.05.

Among the ocular factors, baseline IOP (odds ratio [OR], 0.88; 95% confidential interval [CI], 0.80–0.98; P = 0.014) and percentage reduction of IOP (OR, 0.96; 95% CI, 0.93–0.99; P = 0.020) were associated with recurrent DH in the univariate logistic regression analysis (Table 2).

**Table 2:** Univariate logistic regression analysis of ocular factors.

	Recurrent DH group (n = 89)	Control group (n = 89)	Univariate analysis		
			OR	95% CI	P value
CCT, mean (SD), mm	523.95 (32.52)	526.11 (40.01)	1.00	0.99–1.01	0.736
AXL, mean (SD), μm	24.07 (1.07)	24.36 (1.24)	0.80	0.55–1.16	0.241
Baseline IOP, mean (SD), mmHg	14.92 (3.63)	16.40 (3.35)	0.88	0.80–0.98	**0.014**
IOP with medication, mean (SD), mmHg					
Mean IOP, mean (SD), mmHg	13.51 (2.50)	13.56 (2.22)	0.99	0.85–1.16	0.929
Fluctuation of IOP, mean (SD), mmHg	1.96 (1.50)	1.87 (0.67)	1.10	0.78–1.54	0.588
Maximum IOP, mean (SD), mmHg	16.99 (5.78)	18.03 (4.06)	0.95	0.87–1.04	0.244
Percentage reduction of IOP, mean (SD), %	11.50 (12.81)	17.00 (10.87)	0.96	0.93–0.99	**0.020**
Initial VF MD, mean (SD), dB	-4.15 (5.34)	-4.33 (4.07)	1.01	0.95–1.07	0.806
Initial VF PSD, mean (SD), dB	5.35 (4.33)	5.72 (3.87)	0.98	0.91–1.05	0.541
Average RNFL thickness, mean (SD), μm	90.31 (13.74)	87.27 (13.47)	1.02	0.99–1.05	0.226

DH, disc hemorrhage; OR, odds ratio; CI, confidence interval; SD, standard deviation; CCT, central corneal thickness; AXL, axial length; IOP, intraocular pressure; VF, visual field; MD, mean deviation; PSD, pattern standard deviation; RNFL, retinal nerve fiber layer.

Univariate logistic regression analysis; bolded values represent significance, P < 0.05.

Among the systemic factors, cold extremities (OR, 2.80; 95% CI, 1.03–7.60; P = 0.043), a prone or lateral decubitus sleeping position (OR, 2.14; 95% CI, 1.13–4.03; P = 0.019), and sleep disorder (OR, 2.33; 95% CI, 1.05–5.15; P = 0.037) were associated with recurrent DH in the same univariate logistic regression analysis (Table 3).

**Table 3:** Univariate logistic regression analysis of systemic factors.

	Recurrent DH group (n = 89)	Control group (n = 89)	Univariate analysis		
			OR	95% CI	P value
Diabetes, No (%)	15 (16.9)	15 (16.9)	1.00	0.46–2.19	1.000
Systemic hypertension, No (%)	36 (40.4)	26 (29.2)	1.65	0.88–3.07	0.117
Cardiac disease, No (%)	11 (12.4)	13 (14.6)	0.82	0.35–1.95	0.661
Aspirin or anticoagulant, No (%)	17 (19.1)	18 (20.2)	1.07	0.51–2.25	0.850
Steroid use, No (%)	1 (1.1)	1 (1.1)	1.00	0.06–16.24	1.000
Gingko extract, No (%)	3 (3.4)	2 (2.2)	1.52	0.25–9.31	0.652
Ocular operation history, No (%)	20 (22.5)	21 (23.6)	0.94	0.47–1.89	0.859
Ocular trauma history, No (%)	0	1 (1.1)	1.00	0	1.000
Cold extremities, No (%)	15 (16.9)	6 (6.7)	2.80	1.03–7.60	**0.043**
Migraine, No (%)	7 (7.9)	5 (5.6)	1.43	0.44–4.70	0.552
Glaucoma family history, No (%)	8 (9.0)	8 (9.0)	1.00	0.36–2.79	1.000
Sleeping position (prone/ lateral decubitus), No (%)	38 (42.7)	23 (25.8)	2.14	1.13–4.03	**0.019**
Yoga, No (%)	5 (5.6)	4 (4.5)	1.27	0.33–4.87	0.733
Sleep disorder (sleep apnea/snoring), No (%)	22 (24.7)	11 (12.4)	2.33	1.05–5.15	**0.037**
Constipation, No (%)	2 (2.2)	1 (1.1)	2.02	0.18–22.72	0.568
Heavy load, No (%)	4 (4.5)	6 (6.7)	0.65	0.18–2.39	0.518
Wind instrument playing, No (%)	1 (1.1)	1 (1.1)	1.00	0.06–16.24	1.000

DH, disc hemorrhage; OR, odds ratio; CI, confidence interval.

Univariate logistic regression analysis; bolded values represent significance, P < 0.05.

According to both ocular and systemic factors in our multivariate logistic regression analysis, percent reduction of IOP (OR, 0.96; 95% CI, 0.93–1.00; P = 0.046) was associated with recurrent DH (Table 4).

**Table 4:** Multivariate logistic regression analysis of ocular and systemic factors.

	Model 1			Model 2		
	OR	95% CI	P value	OR	95% CI	P value
Baseline IOP mmHg	1.01	0.85–1.21	0.899			
Percent reduction of IOP %	0.97	0.92–1.01	0.181	0.96	0.93–1.00	**0.046**
Cold extremities No	3.21	0.73–14.18	0.123	3.52	0.82–15.11	0.090
Sleeping position (prone/lateral decubitus) No	1.96	0.80–4.83	0.142			
Sleep disorder (sleep apnea/ snoring) No	1.83	0.61–1.65	0.279			

OR, odds ratio; CI, confidence interval.; IOP, IOP, intraocular pressure

Multivariate logistic regression analysis; bolded values represent significance, P < 0.05

Model 1 includes all of the risk factors with p < 0.10 in univariate analysis

Model 2 includes risk factors selected by backward stepwise method started with items in model 1

### Glaucoma progression

**Fig 1:**
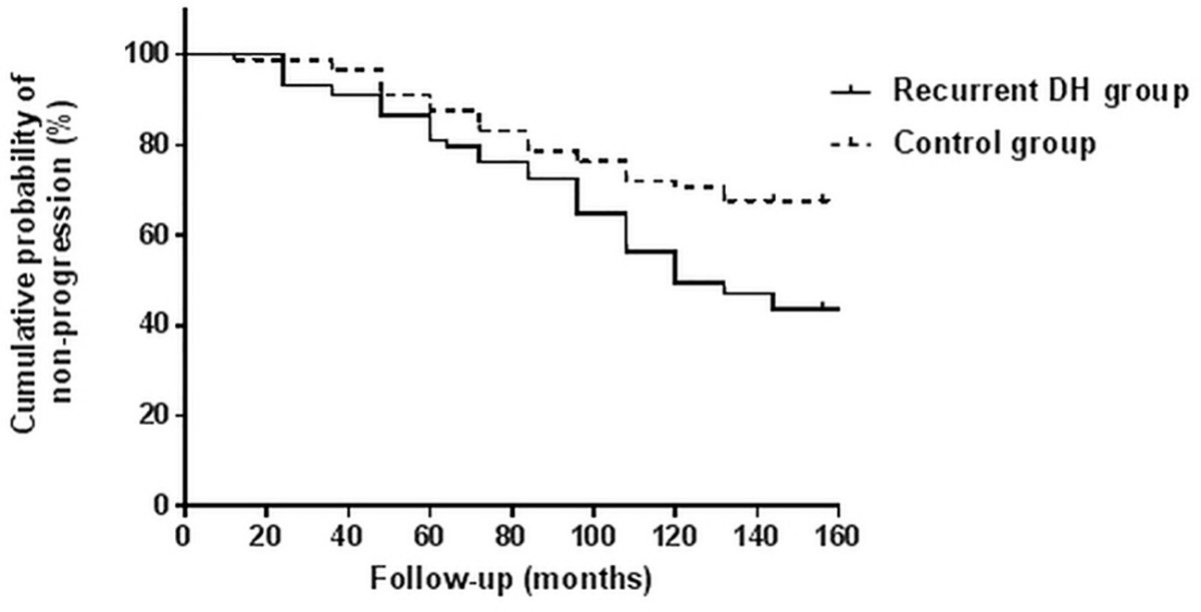
Kaplan-Meier curves comparing cumulative non-progression probability between the recurrent DH and control groups. The statistical endpoint was defined as the time of the first indication of structural or functional glaucoma progression. The control group had a greater cumulative non-progression probability than the recurrent DH group (P = 0.01, log-rank test).

The Kaplan-Meier survival analysis revealed that patients in the control group had a higher probability for cumulative non-progression than patients in the recurrent DH group (P = 0.01, log-rank test) (Fig 1).

The multivariate Cox proportional-hazards regression models revealed that recurrent DH was significantly associated with glaucoma progression (hazard ration (HR), 1.88, 95% CI, 1.16–3.05; P = 0.01) (Table 5).

**Table 5:** Univariate and multivariate Cox proportional hazards regression models’ data for the prediction of progression.

	Univariate			Multiple					
				Model 1			Model 2		
	HR	95% CI	P value	HR	95% CI	P value	HR	95% CI	P value
Baseline IOP	0.99	0.91–1.07	0.72						
Percentage reduction of IOP	0.97	0.95–1.00	0.06	0.98	0.95–1.01	0.12			
Initial VF MD	0.98	0.94–1.03	0.41						
Initial VF PSD	1.05	0.99–1.10	0.08	1.08	1.02–1.15	0.01	1.05	1.00–1.10	0.06
Recurrent DH	1.87	1.15–3.02	**0.01**	2.27	1.22–4.23	0.01	1.88	1.16–3.05	**0.01**
Age	1.00	0.98–1.02	0.73						
Sex	1.30	0.81–2.08	0.27						
Diabetes	1.29	0.68–2.26	0.49						
Hypertension	0.74	0.44–1.24	0.26						
Cold extremities	1.69	0.90–3.14	0.10						
Sleeping position (prone/ lateral decubitus)	0.88	0.52–1.47	0.62						
Sleep disorder (sleep apnea/snoring)	0.95	0.52–1.74	0.88						

HR, hazard ratio; CI, confidence interval; IOP, intraocular pressure; VF, visual field; MD, mean deviation; PSD, pattern standard deviation; DH, disc hemorrhage

Multivariate Cox proportional hazards regression model; bolded values represent significance, P < 0.05

Model 1 includes all of the risk factors with p <0.10 in univariate analysis

Model 2 includes the risk factors selected by backward stepwise method started with items in model 1

## Discussion

The results of our evaluation of the risk factors for recurrent DH in POAG patients demonstrated an association with lower percent reduction of IOP. The strength of our study is its narrow definition of recurrent DH. In the recurrent DH group, we included only eyes showing an occurrence of DH of at least 3 times (recurrent DH) during the follow-up period, and in the control group, only eyes that did not show DH over the course of at least 10 years of follow-up were included. Note that previous studies compared recurrent-DH (with an occurrence of DH of at least 2 times) eyes and no-DH eyes or between recurrent-DH eyes and single-DH eyes [7, 10–12]. Our more strict criteria for the DH and control groups were formulated for the purposes of more clearly isolating the risk factors for the occurrence of DH.

The relationship between IOP and DH remains controversial [6, 13–15]. Soares et al. [16] reported IOP at first DH detection to be lower than the mean IOP of the three most recent visits; and, Jonas et al. [17] suggested that eyes with high IOP had the possibility of earlier bleeding stops. However, Miyake et al. [18] noted that glaucoma patients showed a decreased incidence of DH after trabeculectomy. To our knowledge, ours is the first study to evaluate the association between IOP-related factors such as percent reduction of IOP and recurrent DH. In our results, a lower percent reduction of IOP was the risk factor for recurrent DH. From this, we deduced that IOP reduction is essential for DH-rate reduction. However, we still do not understand the underlying mechanism behind the association between recurrent DH and IOP reduction. One possibility is ischemic microinfarction in the optic disc or the mechanical rupture of small blood vessels caused by IOP-change-related structural alterations in the lamina cribrosa. Nonetheless, further study is required to uncover the pathophysiology of DH and the mechanism behind the correlation between IOP reduction and recurrent DH.

As indicated in Table 5, a prone or lateral decubitus sleeping position was not an associated factor in the multivariate analysis. However, it was an associated factor in the univariate analysis (Table 3). This might be due to the relationship between sleeping position and IOP. Recently, several studies have reported that in the lateral decubitus position, IOP was higher in the dependent eye (lower eye) than in the non-dependent eye (upper eye) [19–21]. Furthermore, among the various sleeping positions, IOP is known to be highest in the prone position. Previously, we reported that no preferred sleeping position (i.e., neither of the one-sided positions) was specifically associated with unilateral DH in normal-tension glaucoma (NTG) [22].

A number of previous studies have evaluated variable associations with DH in glaucoma patients and have suggested several risk factors [5, 16, 23]. However, no study investigating the risk factors for recurrent DH has been conducted. The most frequently cited risk factors are diabetes, systemic hypertension, increasing IOP, and the use of aspirin. However, in our study, diabetes, systemic hypertension, and the use of aspirin were found to be unrelated to recurrent DH. None showed a significant relationship with recurrent DH, even in the univariate logistic regression analysis. With regard to the effect of aspirin on DH, a number of studies have investigated this relationship. Recently, Shim et al. [24] found a significant association between platelet function and DH in glaucoma patients. According to them, that aspirin prevents thromboxane A2 production, which is known to promote platelet aggregation, and this mechanism, through the use of aspirin, can both increase the risk of DH and delay its absorption. However, use of aspirin was not a related factor in our study. Also, it should be noted that the effect of aspirin on platelet function can vary according to dosage levels.

IOP reduction and DH recurrence may be related, but it is difficult to assume a causal relationship. However, in this study, we performed a logistic regression analysis with various ocular and systemic factors to determine which ones might be associated with recurrent DH. According to the results, DH was a relevant factor. We attempted to control the IOP of the patients both with and without DH. Nevertheless, we found that recurrent DH was more common in patients with relatively low IOP reductions. This suggests that IOP might be a risk factor for recurrent DH. In addition, this study demonstrated that recurrent DH was also associated with the progression of glaucoma. This confirms that the IOP reduction is clinically significant for the prevention of glaucoma progression.

Our study has a number of limitations. First, we might have missed some DH cases that had ‘developed and absorbed’ between visits. However, to overcome this limitation, we included, as the control group, only patients who had at least 10 years of follow-up. Moreover, we previously reported that most cases of DH were detected within 5 years of follow-up and that there was no detection-rate increase thereafter [25]. Therefore, the likelihood that we missed any DH incidences is quite low. In addition, recurrent DH was defined as an interval between DH onset and a period of at least 6 months. By doing so, we prevented the erroneous recording of cases of DH non-absorbance as additional DH occurrences. Second, the recurrent DH group exhibited a longer follow-up time and more frequent follow-ups than the no-DH group. One of the reasons for the shorter follow-up interval for the DH group was that those patients were frequently followed-up after the detection of DH. However, once it had been confirmed that DH was absorbed, the follow-up was not significantly different from no-DH cases. Furthermore, the follow-up period was shorter in the recurrent DH group than the control group. However, a mean of 126 months is considered to be a sufficient period of time for the effect of glaucoma medication to manifest. Third, some factors, such as diabetes control, can change over time. The difference in the follow-up period between the two groups might, therefore, be a factor affecting the evaluation of some factors. To minimize this limitation, we excluded patients who had systemic factor changes referenced in their medical records and in the telephone surveys. Fourth, there is theoretical and clinical support for a relationship between baseline IOP and the ability to reduce IOP with treatment [26–28], and it can be difficult to separate the effect of these two variables. Thus, in this study, the multicollinearity between baseline IOP and percent reduction of IOP was assessed by calculating the VIFs. In the results, the VIF value was 1.001 for both baseline IOP and the percent reduction of IOP. This finding suggests that there is no multicollinearity between baseline IOP and percent reduction of IOP. Fifth, the telephone survey itself has limitations. This tool depends on respondents’ memories and possibly subjective answers, and, as such, there is always a possibility that they might not completely or clearly understand their systemic conditions. Additionally, the possibility of such a mild disease being unrecognized by patients is a limitation of most studies conducted via telephone survey. Sixth, trauma history and steroid use were analyzed with only a few data points, so the predictive power may be weak. Seventh, some of the systemic factors used in this study, such as constipation, carrying heavy loads, and wind-instrument playing, can have ambiguous definitions.

In conclusion, our study demonstrates that POAG patients with a lower percent reduction of IOP were at higher risk for the development of recurrent DH. Among ocular and systemic factors, only lower percent reduction of IOP was associated with recurrent DH. This finding suggests that less IOP reduction from baseline might be associated with the recurrence of DH in POAG.

## Supporting information

S1 Table Glaucoma progression. (XLSX)
